# EMT-independent detection of circulating tumor cells in human blood samples and pre-clinical mouse models of metastasis

**DOI:** 10.1007/s10585-020-10070-y

**Published:** 2021-01-07

**Authors:** Jenna Kitz, David Goodale, Carl Postenka, Lori E. Lowes, Alison L. Allan

**Affiliations:** 1 London Regional Cancer Program, London Health Sciences Centre, London, Canada; 2 Department of Anatomy & Cell Biology, Western University, London, Canada; 3 Flow Cytometry, London Health Sciences Centre, London, Canada; 4 Department of Oncology, Western University, London, Canada; 5 Lawson Health Research Institute, London, ON Canada

**Keywords:** Circulating tumor cells, Metastasis, Epithelial-to-mesenchymal transition, CellSearch®, Parsortix®, VyCap

## Abstract

Circulating tumor cells (CTCs) present an opportunity to detect/monitor metastasis throughout disease progression. The CellSearch® is currently the only FDA-approved technology for CTC detection in patients. The main limitation of this system is its reliance on epithelial markers for CTC isolation/enumeration, which reduces its ability to detect more aggressive mesenchymal CTCs that are generated during metastasis via epithelial-to-mesenchymal transition (EMT). This Technical Note describes and validates two EMT-independent CTC analysis protocols; one for human samples using Parsortix® and one for mouse samples using VyCap. Parsortix® identifies significantly more mesenchymal human CTCs compared to the clinical CellSearch® test, and VyCap identifies significantly more CTCs compared to our mouse CellSearch® protocol regardless of EMT status. Recovery and downstream molecular characterization of CTCs is highly feasible using both Parsortix® and VyCap. The described CTC protocols can be used by investigators to study CTC generation, EMT and metastasis in both pre-clinical models and clinical samples.

**Supplementary Information:**

The online version contains supplementary material available at 10.1007/s10585-020-10070-y.

## Introduction

Cancer is the second leading cause of death in the United States; with over 600,000 Americans dying from this disease in 2020 [1]. It is estimated that up to 90% of cancer-related deaths are due to metastasis, the spread of disease to other sites in the body [2]. This is because current therapies are non-curative against these aggressive cancers. The process of metastasis has been shown to be associated with an epithelial-to-mesenchymal transition (EMT) [3]. During the EMT process, a polarized epithelial cell undergoes morphological and molecular changes that enable it to gain a mesenchymal phenotype [4]; characterized by a greater migratory capacity, increased invasiveness, and elevated resistance to apoptosis [5]. During metastasis and associated EMT, tumor cells can shed from the primary tumor and disseminate throughout the body as circulating tumor cells (CTCs) in the bloodstream [6]. The presence and molecular characteristics of CTCs in patients have been correlated with increased metastatic disease, reduced survival, and therapy response/resistance [7–10].

Although EMT has been shown to be associated with increased metastasis and CTC generation, many technologies used to detect CTCs rely on epithelial characteristics [11]. For example, CellSearch® (Menarini Silicon Biosystems) is currently the only CTC assay approved by the U.S. Food and Drug Administration for clinical CTC analysis [7, 8, 12]. CellSearch® distinguishes CTCs from leukocytes through immunomagnetic selection of cells with an EpCAM^+^ (epithelial cell adhesion molecule) phenotype followed by differential fluorescent staining for cytokeratins (CK) 8/18/19, CD45 (leukocyte marker), and DNA (4′,6-diamidino-2-phenylindole [DAPI]). Despite being considered the “gold standard” clinical CTC platform [7, 8, 12], previous studies have shown that in some diseases such as prostate cancer, CTCs are undetectable in ~ 30% of patients despite the presence of widespread metastatic disease [13]. While it is possible that CTCs are truly not present in one third of prostate cancer patients with metastasis, it is more likely that CTCs are present but not detected by the CellSearch® system. This may be because they do not meet the standard CTC definition (EpCAM^+^/CK^+^/DAPI^+^/CD45^−^) due to EMT and associated downregulation of epithelial markers [14, 15].

Importantly, several studies have demonstrated that CTCs with a purely mesenchymal phenotype are undetectable by CellSearch®, but that the presence of mesenchymal marker expression on CTCs with a hybrid epithelial-mesenchymal phenotype is indicative of poor prognosis [15–19]. We have previously described the use of this epithelial-based system in capturing both human and mouse CTCs [20, 21] and demonstrated that a CellSearch®-based assay failed to detect a significant number (~ 40–50%) of mesenchymal CTCs. Notably, the CellSearch®-based assay captured the majority of CTCs shed during early-stage disease in vivo, and only after the establishment of metastases were a significant number of undetectable CTCs present [11]. Taken together, this suggests that current clinical assays may be limiting our ability to capitalize on the full potential of CTCs, and that additional technologies that do not rely on epithelial characteristics should be explored.

The Parsortix® system (Angle PLC) is a sized-based microfluidics platform that allows for recovery of relatively pure populations of CTCs for downstream molecular analysis based on CTC size and deformability, and is thus independent of EMT status [22]. Whole blood is processed through a filtration cartridge etched with microchannels that are 6.5–10 μM wide [20]. Using microfluidics, CTCs (> 8 μM) are isolated within the cartridge and stained with immunofluorescent antibodies [20]. The VyCap system (VyCap B.V.) is a sized-based CTC isolation and enumeration platform which uses a pump unit to process whole blood through a disposable filter cartridge [23, 24]. CTCs are captured on top of the microsieve which has 160,000 pores; each 5 μM in diameter [23, 24]. The VyCap allows for recovery of CTCs based on CTC size rather than epithelial cell characteristics [24] and is thus similar to the Parsortix® in providing the potential for an EMT-independent approach to CTC capture and analysis.

The purpose of this Technical Note is to describe and validate two EMT-independent CTC isolation/enumeration protocols that we have developed for unbiased analysis of CTCs in human blood samples (using Parsortix®) and pre-clinical mouse models of metastasis (using VyCap). We also provide a summary of advantages/disadvantages and technical considerations that metastasis researchers may find valuable for application of these methods to studies in the areas of CTCs, EMT, and cancer progression.

## Materials and methods

### Cell culture and labeling

Epithelial human MDA-MB-468 [25] breast cancer cells (American Type Culture Collection [ATCC], Manassas, VA) were cultured in minimum essential medium (MEM)-α + 10% fetal bovine serum (FBS). Mesenchymal human PC-3 [26] prostate cancer cells (ATCC) were cultured in F12-K media + 10% FBS. Cell lines were authenticated via third-party testing (IDEXX, Columbia, MO). Media and reagents were obtained from Life Technologies (Carlsbad, CA), and FBS from Sigma (St. Louis, MO). For baseline recovery experiments, MDA-MB-468 cells were stained with the CellTrace™ carboxyfluorescein succinimidyl ester (CFSE) Cell Proliferation Kit (Invitrogen, Waltham, MA). Dimethyl sulfoxide (DMSO; 18 µL) was added to one CellTrace™ tube. Dissolved CellTrace™ was added directly to cells suspended in phosphate-buffered saline (PBS) at a concentration of 1:1000. Cells + CellTrace™ were incubated for 20 min at 37 ºC, 5% CO_2_. After incubation, an equal amount of cell culture media was added to the mixture to stop the staining reaction and cells were incubated for a further 5 min. Cells were centrifuged, supernatant was discarded, and cells were resuspended in PBS for counting and spiking into whole blood as described below.

### Blood collection and tumor cell spiking

For human subjects, 2 × 10 mL of whole blood was collected in CellSave preservation tubes (Menarini Silicon Biosystems, Huntingdon Valley, PA). For mice, whole blood (150 µL) was drawn from male athymic nude mice (Harlan Sprague- Dawley, Indianapolis, IN) via cardiac puncture at endpoint as previously described [11]. Blood was collected into ethylenediaminetetraacetic acid (EDTA) microtubes (Becton Dickinson, Mississauga, ON) and separated into two aliquots of 50 µL to be analyzed by each CTC assay. For cell spiking and recovery experiments, unlabeled or prelabeled PC-3 and MDA-MB-468 cells were grown to approximately 80% confluence and harvested using either 0.25% Trypsin/EDTA or 0.25% Trypsin (ThermoFisher Scientific, Waltham, MA) respectively. Cells were counted by hemocytometer and serially diluted using PBS to concentrations of 1000, 100, 10, or 5 cells/10 µL prior to spiking into matched whole blood samples (7.5 mL human; 50µL mouse).

### CTC analysis

#### CellSearch®

For human samples, 7.5 mL of whole blood was processed on the CellSearch® Autoprep system using the CellSearch® CTC kit (Menarini Silicon Biosystems), analyzed on the CellSearch® Analyzer, and assessed for the presence of CTCs as previously described [11]. For mouse samples, 50 µL of whole blood was incubated with components of the CellSearch® CTC kit including anti-EpCAM ferrofluid (25 µL), Capture Enhancement Reagent (25 µL), Nucleic Acid Dye (50 µL), Staining Reagent (50 µL), Permeabilization Reagent (100 µL), as well as anti-mouse CD45-APC (1.5 µL) (eBiosciences, San Diego, CA) as described previously [11]. Samples were manually immuno-magnetically separated and transferred to a CellSearch**®** MagNest™ cartridge for analysis using the CellSearch® Analyzer. In all cases, cells displaying the phenotype of EpCAM^+^/CK^+^/DAPI^+^/CD45^−^ cells with a round/oval morphology were classified as CTCs.

#### Parsortix®

Whole human blood (7.5 mL) was processed on the EMT-independent Parsortix® using 6.5 µM cartridges (Angle PLC, Surrey, UK). Cartridges were stained using a combination of 20 µL anti-human EpCAM-PE (Becton Dickinson), 10 µL anti-human N-Cadherin-PE (eBiosciences), 20 µL anti-human CD45 AlexaFluor-488 (Becton Dickinson), and 5 µL of DAPI (Life Technologies). Cells displaying the phenotype of EpCAM^+^/DAPI^+^/CD45^−^ or N-Cadherin^+^/DAPI^+^/CD45^−^ with a round intact morphology were considered CTCs. Identified CTCs were manually counted on the cartridge using an AX70 microscope (Olympus, Tokyo, JA).

#### VyCap

For the EMT-independent VyCap CTC assay, 3.5 µL of Transfix (Caltag MedSystems, Buckingham, EN) was added to each spiked 50 µL mouse blood sample and incubated at room temperature for 24–48 h. Samples were then incubated for 20 min each with a primary monoclonal anti-human HLA anti-FITC antibody (5 µL, Sigma, Darmstadt, DE), followed by a secondary oligoclonal anti-rabbit unconjugated anti-FITC antibody (5 µL, Thermofisher), tertiary goat anti-rabbit IgG secondary AlexaFluor-488 antibody (5 µL, Invitrogen), and monoclonal anti-mouse CD45-PE antibody (10 µL, Invitrogen) with 2× washing with PBS + 0.5% BSA (BioShop LifeScience Products, Burlington, ON) between each antibody step. Samples were then processed through the VyCap microsieve on the PU-250 pump unit (VyCap, Enschede, NL). Vectashield (5 µL) antifade mounting media with DAPI (Vector Laboratories, Burlingame, CA) was added to the top and bottom of each microsieve prior to being covered with custom cover glass slips (VyCap). Cells displaying the phenotype of HLA^+^/DAPI^+^/CD45^−^ cells with intact round morphology were considered to be CTCs. Identified CTCs were manually counted on the microseives using an AX70 microscope and an LUCPLFLN UPlanFLN 20× Microscope Objective (Olympus).

### In vivo metastasis studies

Prostate cancer cells were prepared in sterile Hank's buffered saline (Life Technologies) and injected (1 × 10^6^ cells/40 µL per mouse) orthotopically into 6–8 week old male athymic nude mice (Harlan Sprague–Dawley) via the right dorsolateral lobe of the prostate as described previously [11]. Prostate cancer tumor growth and progression to metastasis was allowed to develop for 9 weeks. At endpoint, blood (150 µL) was collected and analyzed for CTCs as described above. Tissues (primary tumors and distant organs) were harvested and formalin-fixed, paraffin-embedded, sectioned (4 μm) and stained with hematoxylin and eosin (H&E).

### CTC characterization

#### CTC harvesting

After CTC enumeration on VyCap microseives, coverslips were removed and 50 µL of lysis/binding buffer from the Dynabeads® mRNA Purification Kit (Thermofisher) was added directly onto microsieves. CTCs were lysed via manual pipetting up and down before transfer to 1.5 mL RNAse/DNAse-free microtubes (Diamed, Mississauga, ON). This was repeated twice to ensure total lysis and capture of RNA from all CTCs on each microsieve. For the Parsortix®, cells were collected via the platform’s “harvest protocol” into 1.5 mL RNAse/DNAse-free microtubes (Diamed). CTCs were centrifuged at 700×*g* for 10 min, supernatants were discarded without disturbing the pellet, and CTCs were lysed via manual pipetting using 50 μL lysis/binding buffer as described above. Harvested CTCs in lysis/binding buffer were stored at – 80 ºC prior to analysis as described below.

#### Quantitative real-time PCR analysis

The RNA collected from harvested CTCs was eluted using the Dynabeads® mRNA Purification Kit protocol (Thermofisher) and reverse transcribed using SuperScript™ IV VILO™ Master Mix (Invitrogen) on a T100 Thermal Cycler (BioRad). Samples were then subjected to quantitative real-time polymerase chain reactions (qRT-PCR) using Advanced qPCR MasterMix (Wisent Bioproducts, St.Bruno, QC) on a Stratagene Mx3000P qPCR system (Life Technologies) using primers described in Table 1.

**Table 1:** Forward and reverse primers used for qRT-PCR analysis

Target gene	Forward primer (5′→3′)	Reverse primer (3′→5′)
E-Cadherin	TGCTGATGCCCCCAATACCCCA	GTGATTTCCTGGCCCACGCCAA
EpCAM	CGACTTTTGCCGCAGCTCAGGA	GGGCCCCTTCAGGTTTTGCTCT
N-Cadherin	TGACTCCAACGGGGACTGCACA	AGCTCAAGGACCCAGCAGTGGA
Vimentin	AACCAACGACAAAGCCCGCGTC	TTCCGGTTGGCAGCCTCAGAGA

### Statistical analysis

Statistical analysis was performed using GraphPad Prism 7 for MacOS Mojave (La Jolla, CA). Data is presented as mean ± standard error of the mean (SEM). Paired t-tests were used to analyze differences between matched samples. For all experiments, p ≤ 0.05 was considered statistically significant.

## Results

### The Parsortix® and CellSearch® platforms provide equivalent recovery of epithelial CTCs in human blood samples, but Parsortix® is superior for recovering mesenchymal CTCs

**Fig. 1:**
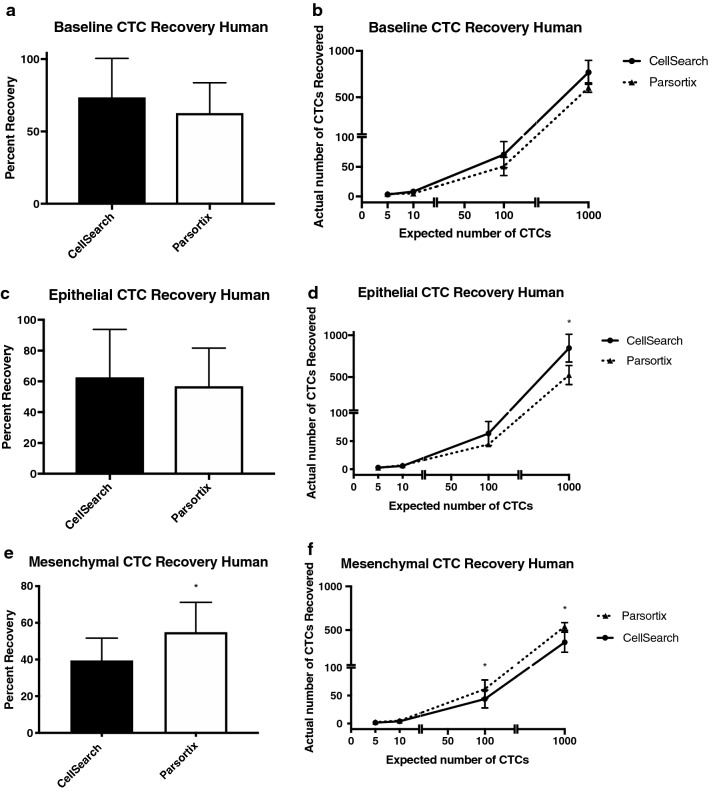
The Parsortix® and CellSearch® CTC platforms provide equivalent recovery of epithelial CTCs in human blood samples, but Parsortix® is superior for recovery of mesenchymal CTCs. Epithelial MDA-MB-468 human breast cancer cells or mesenchymal PC-3 human prostate cancer cells were spiked into whole human blood (5, 10, 100, or 1000 cells per 7.5 ml/blood) and recovered using the human protocols for CellSearch® (epithelial-dependent) or Parsortix® (EMT-independent). **a**, **b** Pre-stained (CellTrace™) epithelial MDA-MB-468 human breast cancer cells spiked into human blood; **c**, **d** Epithelial MDA-MB-468 human breast cancer cells and **e**, **f** Mesenchymal PC-3 human prostate cancer cells spiked into human blood and stained using the human CellSearch® or Parsortix® protocols. Data are presented as mean ± SEM (n ≥ 3), *Significantly different than CellSearch® (p ≤ 0.05)

In order to detect innate differences in capture between the Parsortix**®** and CellSearch® CTC technologies for human samples, we first pre-stained epithelial MDA-MB-468 human breast cancer cells and spiked either 5, 10, 100, or 1000 cells into 7.5 mL of whole human blood. We then enriched for CTCs using the clinical CellSearch® human protocol (with the added GFP channel to identify pre-stained cells), and Parsortix**®** (without the staining protocol) in matched samples. We observed that baseline recovery for CTCs in human blood was not significantly different between the two systems (Fig. 1ab, Table 2). We next wanted to determine differences in CTC recovery in human blood when enumerating epithelial versus mesenchymal CTCs using the clinical epithelial-dependent CellSearch® staining protocol (DAPI^+^/CK-PE^+^/CD45^−^), and our developed epithelial-independent Parsortix® staining protocol (DAPI^+^/EpCAM^+^ or N-Cadherin^+^/CD45^−^). We spiked 5, 10, 100, or 1000 unstained human MDA-MB-468 breast cancer cells (epithelial phenotype) or human PC-3 prostate cancer cells (mesenchymal phenotype) into whole human blood and analyzed CTCs using the two technologies in matched samples. We observed that overall recovery of epithelial CTCs in human blood was not significantly different between the two systems (Fig. 1cd, Table 2), but when assessing cell recovery based on serial numbers of expected cells, CellSearch® was able to enumerate significantly more epithelial CTCs in the cell group of 1000 expected cells compared to Parsortix® (p ≤ 0.05; Fig. 1d, Table 2). However, recovery of mesenchymal CTCs in human blood was significantly higher using Parsortix® (54.9 ± 4.7%) compared to CellSearch® (39.5 ± 3.5%) (p ≤ 0.05; Fig. 1ef, Table 2). Parsortix® was also able to enumerate significantly more CTCs in the 100 and 1000 cell groups compared to CellSearch® (p ≤ 0.05; Fig. 1f, Table 2). Representative images of positive CTCs isolated using CellSearch® and Parsortix® are shown in Online Resource 1. Taken together, these results indicate that while Parsortix® and CellSearch® provide equivalent recovery of epithelial CTCs in human blood samples, Parsortix® is superior for recovery of mesenchymal CTCs.

**Table 2:** CTC recovery in spiked human blood samples using Parsortix**®** versus CellSearch®

Cell type	Number of cells spiked	Cells recovered (Parsortix®)	Cells recovered (CellSearch®)	P-value
Baseline (Pre-stained MDA-MB-468)	5	4.4 ± 0.1	3.3 ± 1.0	0.4226
	10	5.1 ± 1.5	8.0 ± 2.1	0.1994
	100	50.5 ± 7.8	70.4 ± 11.0	0.0989
	1000	604.2 ± 25.4	768.6 ± 64.9	0.1213
Epithelial (MDA-MB-468)	5	2.8 ± 0.45	3.3 ± 1.5	0.6349
	10	7.3 ± 2.4	5.8 ± 0.5	0.6968
	100	44.5 ± 1.6	63.9 ± 10.8	0.2236
	1000	547.7 ± 43.9	846.4 ± 83.6	0.0417*
Mesenchymal (PC-3)	5	2.9 ± 0.7	1.8 ± 0.4	0.2254
	10	4.6 ± 0.9	4.2 ± 0.6	0.2254
	100	61.7 ± 8.5	43.9 ± 8.0	0.0489*
	1000	540.6 ± 23.0	362.0 ± 57.2	0.0473*

*Significantly different between 2 CTC analysis platforms (p ≤ 0.05)

### The VyCap CTC platform provides enhanced recovery of epithelial and mesenchymal CTCs in mouse blood samples compared to CellSearch®

**Fig. 2:**
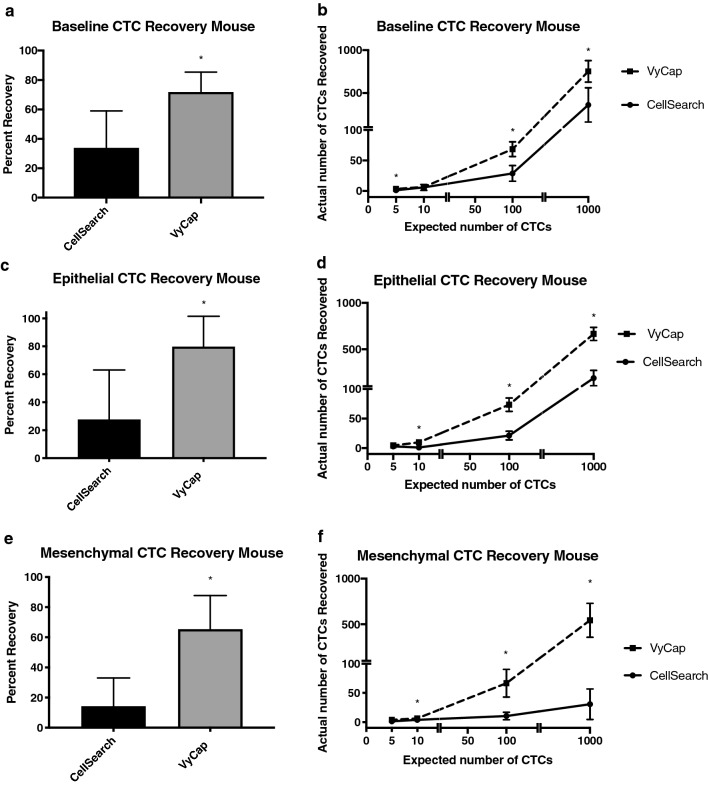
The VyCap CTC platform provides enhanced recovery of spiked-in epithelial and mesenchymal CTCs in mouse blood samples compared to the CellSearch®. Epithelial MDA-MB-468 human breast cancer cells or mesenchymal PC-3 human prostate cancer cells were spiked into whole mouse blood (5, 10, 100, or 1000 cells per 50 µl/blood) and recovered using the mouse protocols for CellSearch® (epithelial-dependent) or VyCap (EMT-independent). **a**, **b** Pre-stained (CellTrace™) epithelial MDA-MB-468 human breast cancer cells spiked into mouse blood; **c**, **d** Epithelial MDA-MB-468 human breast cancer cells and **e**, **f** Mesenchymal PC-3 human prostate cancer cells spiked into mouse blood and stained using the human CellSearch® or VyCap protocols. Data are presented as mean ± SEM (n ≥ 3), *Significantly different than CellSearch® (p ≤ 0.05)

In order to compare CTC capture between the VyCap and CellSearch® technologies for mouse blood samples, we first pre-stained epithelial MDA-MB-468 human breast cancer cells and spiked either 5, 10, 100, or 1000 cells into 50 µL of whole mouse blood. We then enriched for CTCs using our previously developed CellSearch® mouse protocol (with the added GFP channel to identify pre-stained cells), and the VyCap (without the staining protocol) in matched samples. We observed that baseline recovery for CTCs in mouse blood was significantly higher with VyCap (71.9 ± 3.4%) compared to CellSearch® (33.9 ± 6.3%) (p ≤ 0.05; Fig. 2a). When assessing cell recovery based on serial numbers of expected CTCs, VyCap was able to enumerate significantly more CTCs in cell groups of 5, 100, and 1000 expected cells compared CellSearch® in matched samples (p ≤ 0.05); Fig. 2b, Table 3). We next wanted to determine differences in CTC recovery in mouse blood from the perspective of isolation based on an epithelial versus a mesenchymal cell phenotype. To investigate this, we compared the CellSearch® staining protocol (DAPI^+^/CK^−^PE^+^/CD45^−^) versus an epithelial-independent VyCap staining protocol (DAPI^+^/HLA^+^ /CD45^−^) that we developed in-house. We spiked 5, 10, 100, or 1000 unstained human MDA-MB-468 cells breast cancer cells (epithelial phenotype) or human PC-3 prostate cancer cells (mesenchymal phenotype) into whole mouse blood and analyzed CTCs using the two technologies in matched samples. We observed that recovery of epithelial MDA-MB-468 human CTCs in mouse blood was significantly higher using VyCap (79.9 ± 6.2%) compared to CellSearch® (27.7 ± 10.2%) (p ≤ 0.05; Fig. 2c). When assessing cell recovery based on serial numbers of expected cells, VyCap was able to enumerate significantly more epithelial CTCs in cell groups of 10, 100, and 1000 expected cells compared to CellSearch® (p ≤ 0.05; Fig. 2d, Table 3). The difference between CTC platforms was even more marked when assessing the recovery of mesenchymal human CTCs in mouse blood, which was significantly higher using VyCap (65.3 ± 6.5%) compared to CellSearch® (14.3 ± 5.4%) (p ≤ 0.05; Fig. 2ef, Table 3). VyCap was able to enumerate significantly more mesenchymal CTCs in cell groups of 10, 100, and 1000 expected cells compared to CellSearch® (p ≤ 0.05; Fig. 2f, Table 3). Representative images of positive CTCs isolated using CellSearch® and VyCap in each technical condition are shown in Online Resource 2. Taken together, these results indicate that both baseline CTC recovery and overall recovery of CTCs with either an epithelial or mesenchymal phenotype is enhanced through the use of the EMT-independent VyCap system versus the standard CellSearch® protocol.

**Table 3:** CTC recovery in spiked mouse blood samples using VyCap versus CellSearch®

Cell type	Number of cells spiked	Cells recovered (VyCap)	Cells recovered (CellSearch®)	P-value
Baseline (Pre-stained MDA-MB-468)	5	3.9 ± 0.3	1.4 ± 0.8	0.0182*
	10	6.6 ± 1.1	4.3 ± 2.3	0.6971
	100	68.6 ± 7.1	28.7 ± 7.6	0.0042*
	1000	753.0 ± 73.1.6	360.5 ± 116.1	0.0151*
Epithelial (MDA-MB-468)	5	4.1 ± 1.0	3.0 ± 2.0	0.3828
	10	9.8 ± 1.3	1.1 ± 0.6	0.0102*
	100	73.2 ± 6.4	21.4 ± 4.5	0.0026*
	1000	664.7 ± 41.8	187.6 ± 47.7	0.0003*
Mesenchymal (PC-3)	5	3.9 ± 0.8	1.0 ± 0.8	0.0572
	10	6.0 ± 1.3	2.3 ± 1.6	0.0131*
	100	66.7 ± 13.8	10.5 ± 3.8	0.0474*
	1000	566.7 ± 130.1	31.0 ± 15.0	0.0422*

*Significantly different between 2 CTC analysis platforms (p ≤ 0.05)

### The VyCap CTC platform provides enhanced recovery of mesenchymal CTCs from in vivo mouse models of prostate cancer metastasis

**Fig. 3:**
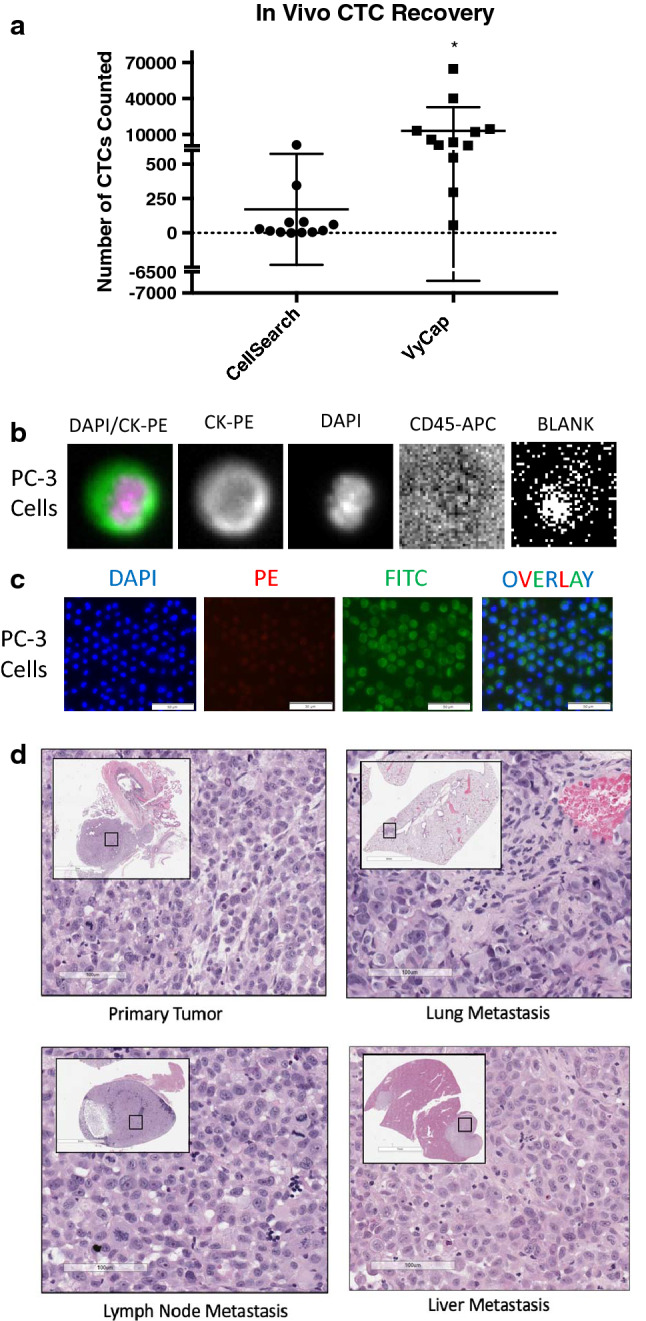
The VyCap CTC platform provides enhanced recovery of mesenchymal CTCs from in vivo mouse models of prostate cancer metastasis*.* PC-3 cells were orthotopically injected into the prostate gland of male nude mice. Prostate cancer tumor growth and progression to metastasis was allowed to develop for 9 weeks. At endpoint, blood was collected and analyzed for CTCs using mouse protocols for CellSearch® (epithelial-dependent) or VyCap (EMT-independent). **a** Recovery of in vivo CTCs by CellSearch® versus VyCap. Data are presented as mean ± SEM CTCs/50 µl of blood/mouse (n = 12), *Significantly different than CellSearch® (p ≤ 0.05). **b** Representative images of positive CTCs isolated using CellSearch®. **c** Representative images of positive CTCs isolated using VyCap. **d** Representative H&E staining of primary tumor and metastatic sites

In order to assess the value of our developed mouse VyCap EMT-independent protocol compared to the mouse CellSearch® protocol in vivo, we orthotopically injected 12 mice with mesenchymal PC-3 human prostate cancer cells. After 9 weeks of primary tumor growth and disease progression, mice were sacrificed, blood samples were collected, and CTCs were enumerated using our two protocols. We observed that the VyCap was able to recover CTCs in all 12 mice, whereas the CellSearch® was only able to capture CTCs in 10/12 mice with metastatic prostate cancer (Fig. 3a–c). The VyCap also provided significantly enhanced recovery of CTCs in mice with metastatic prostate cancer (13,094 ± 5719 CTCs/mouse) compared to CellSearch® (171 ± 117 CTCs/mouse) (p ≤ 0.05; Fig. 3a–c). Of these mice, we observed that 8/12 mice developed metastatic disease in one or more organs as determined by pathohistological analysis (Fig. 3d). In addition to detecting CTCs in the 8 mice with detectable metastases, VyCap was also able to detect a significant number of CTCs in all 4 mice in which metastases were histologically undetectable, although the numbers of CTCs observed were lower. In contrast, the CellSearch® was only able to detect CTCs in 2 of these mice. These results support our observations from the spiking studies and validate our newly developed EMT-independent VyCap protocol for use in pre-clinical mouse studies of CTCs and metastasis.

### CTCs can be harvested from the VyCap and Parsortix® for downstream molecular characterization

**Fig. 4:**
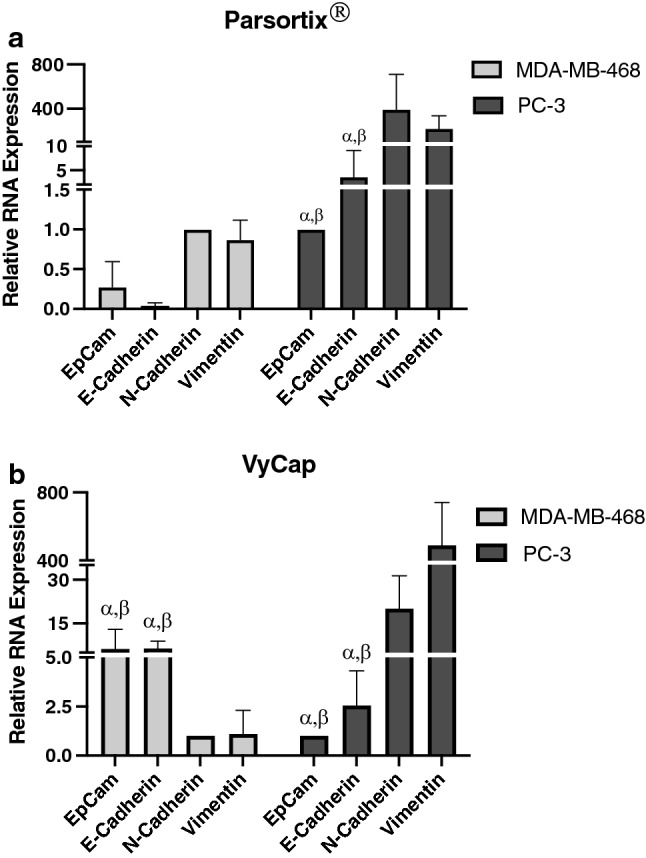
CTCs can be harvested from the VyCap and Parsortix® systems for downstream molecular characterization. Epithelial MDA-MB-468 human breast cancer CTCs or mesenchymal PC-3 human prostate cancer CTCs were enumerated on the **a** Parsortix® or **b** VyCap platforms and CTCs were harvested after enumeration. RNA was isolated from captured CTC samples and assessed for expression of the EMT markers EpCAM, E-Cadherin, N-Cadherin, and Vimentin using qRT-PCR. Data are presented as mean ± SEM (n = 3), *α* = significantly different than N-Cadherin (p ≤ 0.05), *β* = significantly different than Vimentin (p ≤ 0.05)

Finally, we wanted to assess the feasibility of harvesting CTCs from the two EMT-independent platforms and using them for downstream molecular characterization. Following CTC enumeration by either Parsortix® or VyCap, CTCs were harvested and RNA was isolated for qRT-PCR to assess expression of EMT markers in MDA-MB-468 (epithelial) and PC-3 (mesenchymal) CTC samples. Overall, we observed that EMT gene expression could be detected in isolated epithelial or mesenchymal CTCs harvested both platforms, although the expression patterns were more consistent with what was expected using the VyCap (Fig. 4ab; p ≤ 0.05). In particular, MDA-MB-468 CTCs harvested from Parsortix® did not show the expected epithelial gene expression pattern (p > 0.05). These results demonstrate the ability to isolate RNA and characterize gene expression from CTC samples via the VyCap or Parsortix**®** for further downstream characterization after CTC enumeration.

## Discussion

Analysis of CTCs holds tremendous promise for tracking metastatic progression and treatment response in both human cancer patients and pre-clinical mouse models of metastasis. However, current clinical assays such as CellSearch® rely on epithelial cell characteristics for CTC detection and enumeration, and thus may be limiting our ability to capitalize on the full potential of CTCs. In the current study we developed and validated two EMT-independent CTC enumeration and harvest protocols, one for use with human patient samples using the Parsortix® (EpCAM^+^ or N-Cadherin^+^ phenotype), and one for use with pre-clinical mouse samples using VyCap (HLA^+^ phenotype), and compared them to the clinical “gold standard” FDA-approved CellSearch®.

For analysis of human samples, we observed no significant differences in CTC capture for epithelial CTCs between CellSearch® and Parsortix®. Thus, either system may be appropriate for enumeration of epithelial CTCs from human blood samples depending on the study design. For example, studies evaluating early-stage cancers, the initiating steps of metastasis, or epithelial marker expression on CTCs could be carried out using either CellSearch® and Parsortix®. However, in studies of more advanced and/or aggressive cancers where a greater proportion of mesenchymal CTCs or mixed epithelial/mesenchymal CTCs are expected, Parsortix® might be a more appropriate CTC platform based on our observations that significantly enhanced detection of mesenchymal CTCs is possible with Parsortix® versus CellSearch®. For CTC capture in pre-clinical mouse models, our results indicate that our developed VyCap protocol (EMT-independent) is superior to our previously developed mouse CellSearch® protocol (EpCAM-dependent) regardless of cell phenotype. This is likely due to both the epithelial-dependent nature of the CellSearch® platform as well as the manual CTC enrichment step in the CellSearch® mouse protocol (including multiple wash steps) which may cause loss of CTCs during the isolation process [11].

Additionally, the ability to harvest CTCs from the different platforms for downstream analysis is an important consideration when choosing which technology is most appropriate, since CellSearch® does not allow the user to recover enumerated CTCs. Therefore, for investigators interested in tracking evolving molecular characteristics throughout disease progression or assessing expression of specific therapeutic targets, our results suggest that VyCap or Parsortix® may be more appropriate platforms to use compared to CellSearch®. Our results also indicate that the VyCap platform may be slightly more optimal for cell harvesting compared to the Parsortix®, potentially due the differences in isolation procedure. With the VyCap, the RNA lysis buffer is added directly to the microsieve, with full exposure to all CTCs present and potentially improved recovery and RNA extraction. In contrast, with the Parsortix® system, tumor cells move through an increasingly smaller area until they become lodged within the stepwise system of the chip. It is possible that some larger CTCs may become stuck within the chip and are not dislodged by the backflow pressure in the harvest protocol and thus do not get harvested. This may result in an insufficient cell number for RNA extraction and accurate qRT-PCR analysis, especially with low numbers of CTCs. This may be of particular concern when using immortalized cell lines for CTC studies, which have been demonstrated to have a greater diameter in circulation (~ 15–20 µm) than primary patient CTCs (~ 10–13 µm) [27]. Similarly, breast cancer CTCs are typically larger than prostate cancer CTCs [27] which may help explain why we did not obtain the expected epithelial EMT gene expression results from MDA-MB-468 breast cancer cells harvested from the Parsortix®. Thus, recovery of CTCs from the Parsortix® for downstream analysis may be further optimized by careful selection of the most appropriate sized cartridge (6.5, 8 or 10 µm) for the disease site and/or experimental question being investigated.

Each of the three CTC platforms described in this Technical Note have a number of advantages and disadvantages that researchers should consider when designing their CTC studies and choosing an appropriate analysis platform (Table 4). For example, the FDA-approved status and the significant body of clinical prognostic data available for CellSearch® supports its use in clinical studies, particularly those where mostly epithelial CTCs are expected. However, it may potentially miss aggressive mesenchymal CTCs and it provides very limited capacity for recovery and downstream analysis of CTCs. The Parsortix® addresses many of these limitations, and although it is not yet FDA-approved, its potential clinical validity is supported by a CE mark in Europe and a number of promising clinical studies [20]. For example, in a recently completed clinical trial, Parsortix® was successfully used to isolate and harvest CTCs from metastatic breast cancer patients for further downstream analysis in support of an upcoming FDA submission (ClinicalTrials.gov; NCT03427450). Ongoing clinical studies are also using Parsortix® for the isolation of rare CTCs in ovarian cancer (ClinicalTrials.gov; NCT02781272), to evaluate multiple biomarkers in ovarian cancer (ClinicalTrials.gov; NCT02785731), in an EMT-independent prostate cancer study (ClinicalTrials.gov; NCT04021394), and for evaluating heterogeneity and predicting clinical relapse in non-small cell lung carcinoma patients (ClinicalTrials.gov; NCT03771404). Pending FDA approval, the unique attributes of Parsortix® such as easy marker customization and the ability to harvest CTCs for downstream analysis [28, 29] will position Parsortix® as an ideal CTC platform for use in clinical trials and clinical management. However, one of the main limitations of the Parsortix® is the time it takes to process a single sample; approximately seven hours to separate, stain, harvest, and clean the instrument in preparation for the next sample. The low-throughput nature of Parsortix® is challenging but manageable for clinical samples, which typically arrive in the lab one at a time. However, for pre-clinical studies, researchers often have multiple mice in each group with set endpoints or blood collection points. Using the VyCap [23, 30, 31], mouse blood samples can be pre-stained in batches in two hours using custom antibody panels, enriched in less than a minute per sample, and CTC RNA harvested in approximately five minutes off the disposable microsieves. This allows the user to stain, separate, enumerate, and harvest up to twelve samples in one day. Due to the increased sample throughput of the VyCap compared to the Parsortix®, it is a better platform to assess CTCs in pre-clinical mouse experiments where multiple samples need to be collected and analyzed together. With the ability to further enhance analysis capacity through the additional use of VyCap’s semi-automated microscopy system [32] and/or single CTC isolation puncher [33], this system provides a high degree of flexibility for CTC studies. However, similar to the Parsortix®, VyCap does not yet have FDA approval for clinical use and has not yet demonstrated clinical validity in terms of association with patient prognosis or response to treatment; and this is the major limitation of this system [34].

**Table 4:**
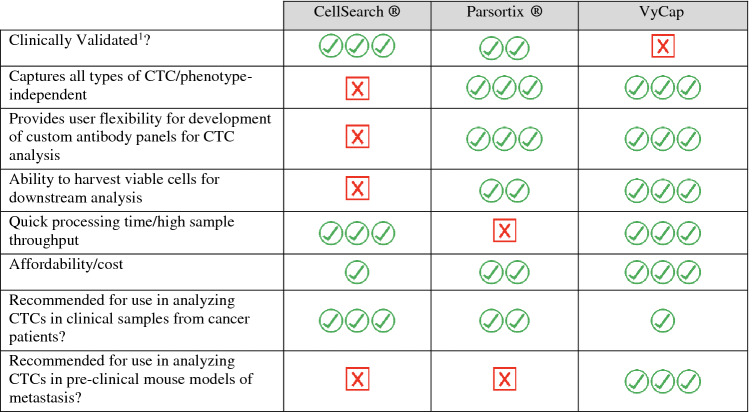
Advantages and disadvantages of CellSearch®, Parsortix® and VyCap CTC analysis platforms

^1^Based on regulatory approval and/or published clinical data demonstrating an association with patient prognosis and/or response to therapy

In summary, we believe that this Technical Note will be valuable for aiding researchers in decision-making regarding which CTC platform is best for their specific studies. Taken together, this will help enhance knowledge in the areas of CTC generation, metastasis, and EMT to ultimately assist in treating patients with aggressive metastatic disease.

## Supplementary Information

Below is the link to the electronic supplementary material.

Electronic supplementary material 1 (PDF 257 kb)

Electronic supplementary material 2 (PDF 277 kb)

## Data Availability

The authors agree that data and material described in the published manuscript will be made available upon request.
