# Supplementary material for: EMT-independent detection of circulating tumor cells in human blood samples and pre-clinical mouse models of metastasis
Source: Clin Exp Metastasis. 2021 Jan 7;38(1):97–108. doi: 10.1007/s10585-020-10070-y (PMC7882592; doi:10.1007/s10585-020-10070-y)
Supplement: Supplementary file 1 — Electronic supplementary material 1 (PDF 257 kb) [file 10585_2020_10070_MOESM1_ESM.pdf]

Online Resource 1

CLINICAL & EXPERIMENTAL METASTASIS

EMT-independent detection of circulating tumor cells in human blood samples and pre-clinical mouse models of metastasis

Jenna Kitz[1,2], David Goodale[1], Carl Postenka[1], Lori E. Lowes[3], and Alison L. Allan[1,2,4,5*]

London Regional Cancer Program[1] and Flow Cytometry[3], London Health Sciences Centre; Departments of Anatomy & Cell Biology[2] and Oncology[4], Western University; and Lawson Health Research Institute[5], London, Ontario CANADA

[*]***Corresponding author***; alison.allan@lhsc.on.ca or 519-685-8600 x55134

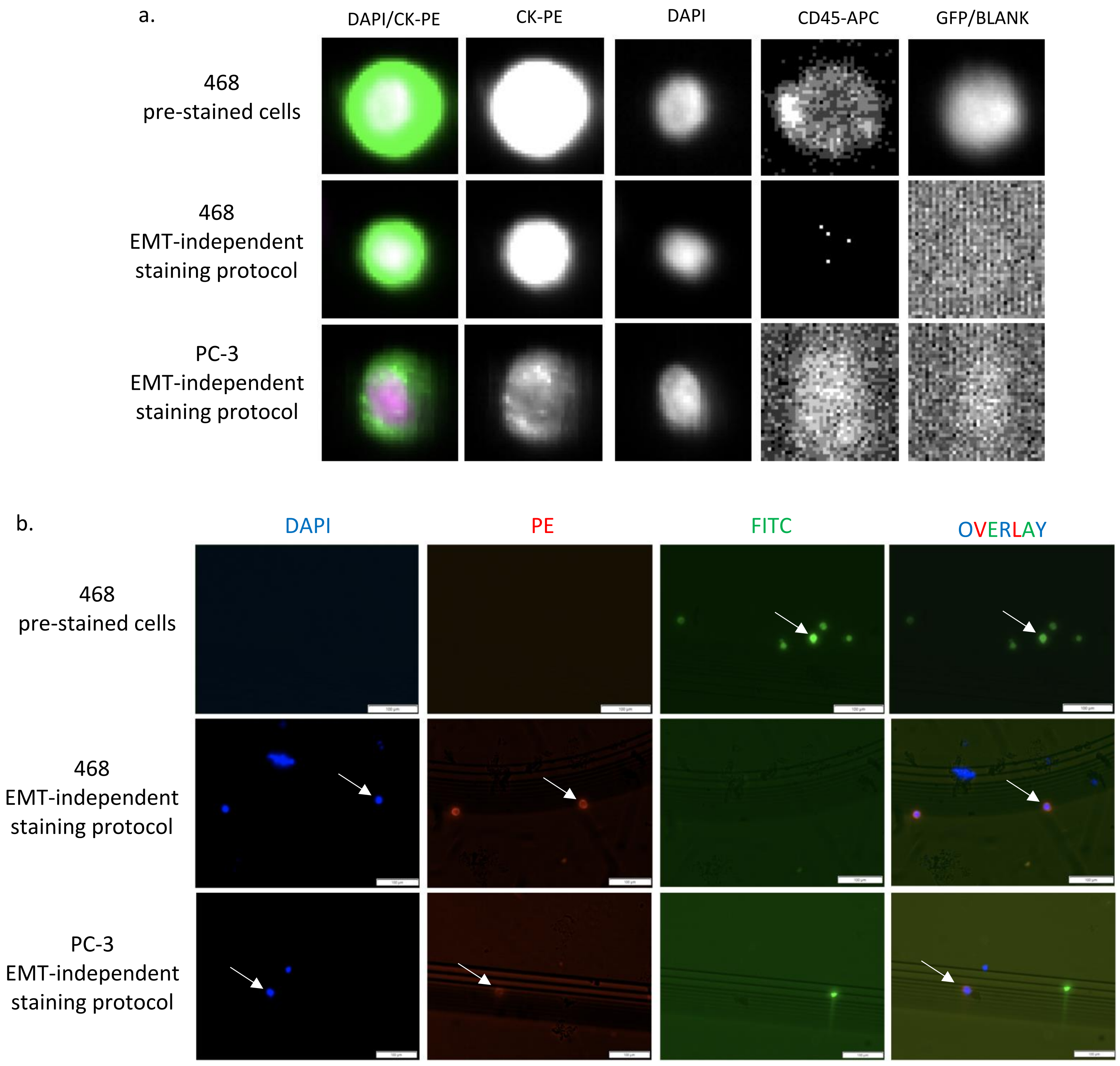

Online Resource 1 *Representative images of positive CTCs isolated from human blood using CellSearch® and Parsortix®*. For baseline recovery experiments, MDA-MB-468 human breast cancer cells (epithelial) were prestained with the CellTrace™ carboxyfluorescein succinimidyl ester (CFSE) Cell Proliferation Kit. For all other experiments, human MDA-MB-468 cells or human PC-3 mesenchymal cells were stained using the human staining protocols outlined in the Materials & Methods. *(a)* Representative positive CTCs isolated using the human CellSearch® protocol. DAPI$^+$/CK-PE$^+$/CD45$^-$/GFP(CFSE)$^+$ cells are considered to be positive CTCs in pre-stained samples. DAPI$^+$/CK-PE$^+$/CD45$^-$ cells are considered to be positive CTCs in samples processed with the CellSearch® CTC kit. *(b)* Representative positive CTCs isolated using the human Parsortix® protocol (*white arrows*). FITC (CFSE)$^+$ are considered to be positive CTCs in pre-stained samples. For samples stained with the EMT-independent staining protocol, DAPI$^+$/PE$^+$/FITC$^-$ cells are considered positive (DAPI (blue) = nuclear stain, PE (red) = EpCAM or N-Cadherin, FITC (green) = CD45). Data are presented as the mean ± SEM ($n \geq 3$), * = significantly different than CellSearch® ($p \leq 0.05$)
