# Supplementary material for: EMT-independent detection of circulating tumor cells in human blood samples and pre-clinical mouse models of metastasis
Source: Clin Exp Metastasis. 2021 Jan 7;38(1):97–108. doi: 10.1007/s10585-020-10070-y (PMC7882592; doi:10.1007/s10585-020-10070-y)
Supplement: Supplementary file 2 — Electronic supplementary material 2 (PDF 277 kb) [file 10585_2020_10070_MOESM2_ESM.pdf]

Online Resource 2

CLINICAL & EXPERIMENTAL METASTASIS

EMT-independent detection of circulating tumor cells in human blood samples and pre-clinical mouse models of metastasis

Jenna Kitz[1,2], David Goodale[1], Carl Postenka[1], Lori E. Lowes[3], and Alison L. Allan[1,2,4,5*]

London Regional Cancer Program[1] and Flow Cytometry[3], London Health Sciences Centre; Departments of Anatomy & Cell Biology[2] and Oncology[4], Western University; and Lawson Health Research Institute[5], London, Ontario CANADA

[*]***Corresponding author***; alison.allan@lhsc.on.ca or 519-685-8600 x55134

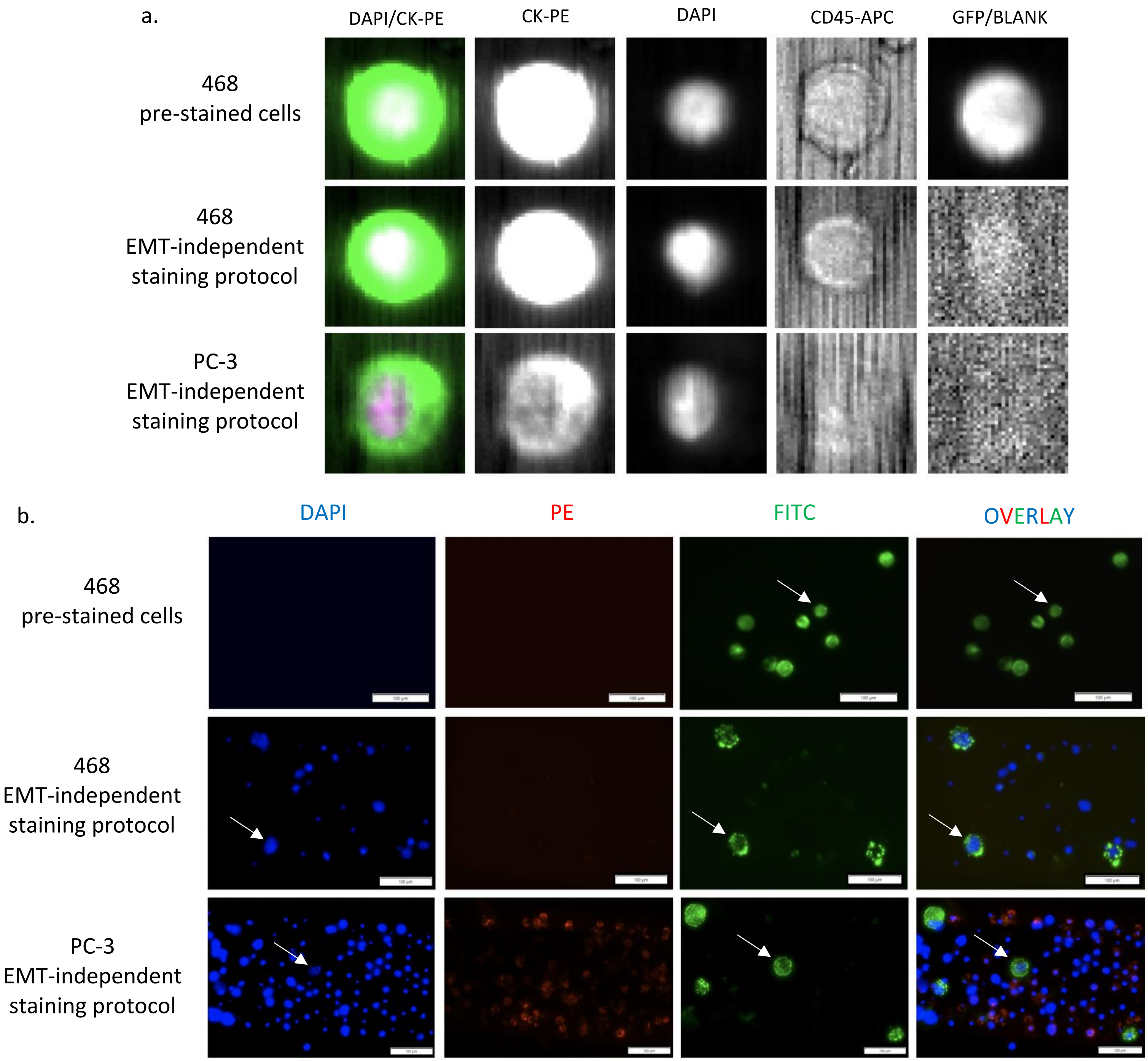

Online Resource 2 *Representative images of positive CTCs isolated from mouse blood using CellSearch® and VyCap.* For baseline recovery experiments, MDA-MB-468 human breast cancer cells (epithelial) were prestained with the CellTrace™ carboxyfluorescein succinimidyl ester (CFSE) Cell Proliferation Kit. For all other experiments, human MDA-MB-468 cells or human PC-3 mesenchymal cells were stained using the mouse staining protocols outlined in the Materials & Methods. *(a)* Representative positive CTCs isolated using the mouse CellSearch® protocol. DAPI$^+$/CK$^-$PE$^+$/CD45$^-$/GFP(CFSE)$^+$ cells are considered to be positive CTCs in pre-stained samples. DAPI$^+$/CK$^-$PE$^+$/CD45$^-$ cells are considered to be positive CTCs in samples processed with the CellSearch® CTC staining protocol. *(b)* Representative positive CTCs isolated using the mouse VyCap protocol (*white arrows*). FITC (CFSE)$^+$ are considered to be positive CTCs in pre-stained samples. For samples stained with the EMT-independent staining protocol, DAPI$^+$/PE$^-$/FITC$^+$ cells are considered positive (DAPI (blue) = nuclear stain, PE (red) = CD45, FITC (green) = HLA). Data are presented as the mean ± SEM ($n \geq 3$), * = significantly different than CellSearch® ($p \leq 0.05$)
